# Characteristics of the Kelch domain containing (KLHDC) subfamily and relationships with diseases

**DOI:** 10.1002/1873-3468.15108

**Published:** 2025-01-30

**Authors:** Courtney Pilcher, Paula Armina V. Buco, Jia Q. Truong, Paul A. Ramsland, Monique F. Smeets, Carl R. Walkley, Jessica K. Holien

**Affiliations:** ^1^ School of Science, STEM College RMIT University Melbourne Australia; ^2^ St Vincent's Institute of Medical Research Fitzroy Australia; ^3^ Department of Medicine, Eastern Hill Academic Centre, Melbourne Medical School The University of Melbourne Carlton Australia; ^4^ Department of Immunology Monash University Melbourne Australia; ^5^ Department of Surgery, Austin Health The University of Melbourne Melbourne Australia; ^6^ Centre for Innate Immunity and Infectious Diseases Hudson Institute of Medical Research Melbourne Australia; ^7^ Department of Molecular and Translational Science Monash University Melbourne Australia

**Keywords:** Kelch family, KLHDC, protein degradation, protein function

## Abstract

The Kelch protein superfamily is an evolutionary conserved family containing 63 alternate protein coding members. The superfamily is split into three subfamilies: Kelch like (KLHL), Kelch‐repeat and bric‐a‐bracs (BTB) domain containing (KBTBD) and Kelch domain containing protein (KLHDC). The KLHDC subfamily is one of the smallest within the Kelch superfamily, containing 10 primary members. There is little known about the structures and functions of the subfamily; however, they are thought to be involved in several cellular and molecular processes. Recently, there have been significant structural and biochemical advances for KLHDC2, which has aided our understanding of other KLHDC family members. Furthermore, small molecules directly targeting KLHDC2 have been identified, which act as tools for targeted protein degradation. This review utilises this information, in conjunction with a thorough exploration of the structural aspects and potential biological functions to summarise the relationship between KLHDCs and human disease.

## Graphical abstract

The Kelch protein superfamily includes 63 members, with the KLHDC subfamily having 10 proteins. While their functions are not fully understood, recent advances in KLHDC2's structure and role in protein degradation have highlighted its potential for drug development, especially in PROTAC therapies. This review explores the structural aspects and biological functions of KLHDCs, linking them to human disease.

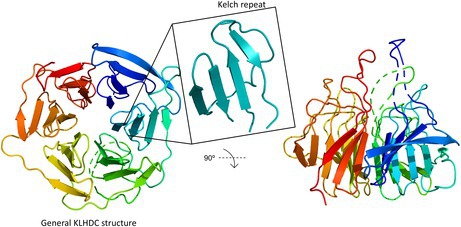

## Abbreviations

**ASK1**, apoptosis signal‐regulating kinase 1

**BACK**, BTB and C‐terminal Kelch

**BTB**, bric‐a‐bracs

**CCT**, chaperonin‐containing TCP1

**CRISPR**, clustered regularly interspaced short palindromic repeats

**Cryo‐EM**, Cryo‐electron microscopy

**CTLH**, alpha‐helical segment, C‐terminal to LiSH

**CUL**, Cullin

**DesCEND**, destruction *via* C‐end degrons

**DNA**, deoxyribonucleic acid

**ELO**, elongin

**ER**, endoplasmic reticulum

**GST**, glutathione S‐transferase

**HGNC**, HUGO Gene Nomenclature Committee

**KBTBD**, Kelch‐repeat and bric‐a‐bracs

**KLHDC**, Kelch domain containing protein

**KLHL**, Kelch like

**LiSH**, Lissencephaly‐1

**MKLN1**, Muskelin 1

**NMR**, nuclear magnetic resonance

**NSCLC**, non–small‐cell lung cancer

**PDP2**, pyruvate dehydrogenase

**POZ**, poxvirus and zinc finger domain

**PP5**, protein phosphatase 5

**PPP1R15A**, protein phosphatase 1 regulatory subunit 15A

**PROTAC**, proteolysis targeting chimeras

**PTOV**, prostate tumour‐overexpressed protein

**RABEPK**, Rab9 effector protein with Kelch motifs

**RBX**, ring‐box protein

**RBX1**, ring‐box protein 1

**RING**, really interesting new gene

**RNA**, ribonucleic acid

**Sec**, selenocysteine

**Sel**, selenoprotein

**SEPHS2**, selenophosphate synthetase 2

**SRR**, substrate recognition receptor

**TCAP**, telethonin

**TSPYL**, testis‐specific Y‐encoded‐like

**Ub**, ubiquitin

**UPS**, ubiquitin proteasomal system

**USP**, ubiquitin‐specific peptidase

**UTR**, untranslated region

## The Kelch protein superfamily

The Kelch domain was first described as a repeat element in the sequence of the *Drosophila* ORF1 protein in 1993 [1]. Since then, multiple Kelch‐repeat proteins have been identified through sequencing a diverse range of organisms including viruses, plants, fungi and mammals [2]. The Kelch domain consists of several Kelch repeats and is one of the largest evolutionary conserved protein folds [1, 2]. Each Kelch‐repeat structure, termed a ‘blade’, consists of three to four twisted antiparallel β‐strands connected by intrablade loops. Five to seven Kelch repeats form the distinct ‘β‐propeller’ of the Kelch domain [3]. Kelch domains function as scaffolds for protein–protein interactions, primarily binding substrates for ubiquitination and subsequent degradation.

**Fig. 1:**
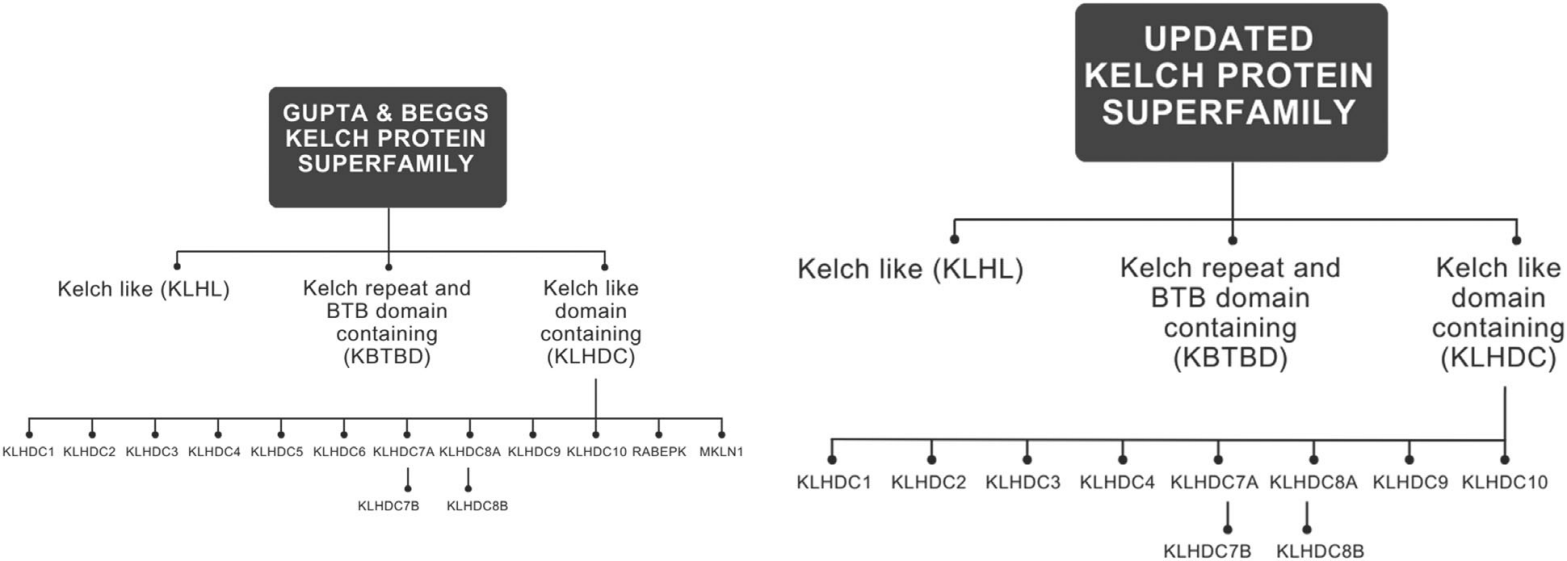
Kelch protein superfamily. There are three subfamilies – Kelch like (KLHL), Kelch‐repeat and bric‐a‐bracs domain containing (KBTBD) and Kelch domain containing protein (KLHDC). Initial phylogenetic analysis suggested 12 members of the KLHDC subfamily (left); however, recent evidence has suggested that there are only 10 subfamily members [95].

The complement of human Kelch proteins was initially defined with 57 Kelch‐repeat proteins. The list was subsequently expanded to include 63 alternate genes for Kelch‐repeat domain containing proteins [1, 2, 3, 4], of which three are non‐protein–coding genes [3]. These were initially grouped into five subgroups [1, 5] defined by the position of the β‐propeller domain and the presence of other protein domains within the primary sequences. However, emerging details surrounding the discovery of BACK domain [6] and review of the KLHL (Kelch‐like) literature [5, 6] ultimately led to the update of this group, as proposed by Gupta and Beggs [3]. This redefined the five subgroups into three – KLHL, KBTBD (Kelch repeat and broad complex, Tram track domain containing) and KLHDC (Kelch domain containing) – based on the number of Kelch repeats in addition to the presence of alternative domains (Fig. 1). Additionally, a phylogenetic analysis of the human Kelch family proteins was also completed by Gupta and Beggs [3], which showed that most members in each of the subfamilies (KLHL, KBTBD and KLHDC) largely clustered together with the KLHDC subfamily, suggesting that KLHDC subfamily diverged first with a gain or loss of Kelch family domains occurring at later stages of evolution. Since this review focuses on the KLHDC family, it will not describe the other domains in detail, but these have been well documented by others [2, 3, 4, 5, 6, 7].

The KLHL subfamily contains all atypical Kelch protein domains, that is, an N‐terminal BTB (bric‐a‐brac, tram track, broad complex)/POZ (poxvirus and zinc finger) domain, a BACK (for BTB and C‐terminal Kelch) [6] domain and between two and eight Kelch repeats. KLHL is known to form a complex with Cullin3 and RBX1 [8, 9, 10], where the BTB/POZ domain is responsible for binding to Cullin3, while the Kelch domain determines the substrate specificity, and the BACK domain links both BTB and Kelch domains [11].

The KBTBD subfamily members typically lack the BACK domain [6], highlighting that these likely diverged in the later stages of evolution [3]. The KLHDC subfamily does not contain either BTB or BACK domains [5, 6]; however, some have alternate domains such as the transmembrane and glycine rich domains [3], which are known to be functionally important.

## Kelch‐like domain containing protein (KLHDC) subfamily

In 2014, Gupta and Beggs [3] proposed a KLHDC subfamily that contained 12 primary members (Fig. 1), with some members having 1–3 additional isoforms (denoted with *) – KLHDC1, KLHDC2*, KLHDC3, KLHDC4*, KLHDC7A and KLHDC7B, KLHDC8A and KLHDC8B, KLHDC9*, KLHDC10, RABEPK and MKLN1, based on a phylogenetic analysis using each of their Kelch domain sequences. Additionally, two more proteins (KLHDC5 and KLHDC6) have also been classified as KLHDC subfamily members previously. However, we propose a revised number of members from 12 to 10 (Fig. 1), with the following justifications. In the case of KLHDC5, experimental data obtained by Cummings *et al*. [12] initially found that KLHDC5 contained a BTB domain; additionally, it was Dhanoa *et al*. [5] who also indicated that based on the structure (presence of BTB, BACK and 5–6 Kelch repeats), KLHDC5 should be reannotated from KLHDC subfamily (KLHDC5) to the KLHL subfamily (KLHL42). Furthermore, these structural findings are also supported by the alphafold prediction server [13], which predicts KLHL42 (Uniprot Entry [14]: Q9P2K6, alphafold [13] entry: AF‐Q9P2K6‐F1) to have both a BTB and BACK domain with ‘very high’ model confidence. Consequently, nomenclature and classification systems, such as the HUGO Gene Nomenclature Committee (HGNC) in the human genome [15] and UniProt [14], have also recognised these updates and have since reclassified KLHDC5 to KLHL42. KLHDC6 is relatively less researched; however, complete sequencing of full length human cDNAs [16], the use of domain prediction software such as interpro ([14, 17]: Q3ZCT8) and some initial alignment and sequence searches revealed KLHDC6 to contain a BTB domain [18]; hence, KLHDC6 has now been reclassified to KBTBD12. Like in the case of KLHL42, the structural findings are also supported by alphafold prediction server [13], which predicts that KBTBD12 (Uniprot entry [14]: Q3ZCT8, alphafold [13]: AF‐Q3ZCT8‐F1) contains a BTB domain with ‘very high’ model confidence. This reclassification is also acknowledged by HGNC [15] and UniProt [14], who have also reflected this change within their online servers.

Finally, while Muskelin 1 (MKLN1) and Rab9 effector protein with Kelch motifs (RABEPK) show initial characteristics that could warrant their inclusion in the KLHDC subfamily – such as the presence of similar structural domains (Kelch‐repeat motifs) – further evidence, such as an up to date and detailed phylogenetic and structural analysis to confidently classify them within the KLHDC subfamily, is severely lacking. MKLN1 contains both a LiSH (Lissencephaly‐1 homology – an alpha helical region of approximately 60 amino acids at the N terminus) motif and a CTLH (alpha‐helical segment, C‐terminal to LiSH) motif [19]. Additionally, experimental data [19] identified an N‐terminal region as a predicted discoidin‐like domain, making it the only Kelch‐repeat protein identified in the human genome to possess a discoidin domain [2]. The presence of these motifs and domains distinguishes MKLN1 from other Kelch‐repeat proteins [19]. Furthermore, through sequence identity comparison of MKLN1 and the rest of the KLHDC subfamily, MKLN1 has the lowest percent identity (average ~ 16%) (Table 1), and thus, we believe that MKLN1 cannot be classified at this time specifically within the KLHDC subfamily. Similarly, RABEPK also shares a low percent identity (average ~ 19%) (Table 1), which is likely reflective of the presence of the Kelch‐repeat motifs; however, this suggests weak evolutionary conservation. Additionally, the functional role of RABEPK diverges significantly to that of the KLHDC subfamily, with it primarily acting as an effector protein for Rab9, a small GTPase involved in vesicle transport [20, 21]. This has little overlap with the functions typically associated with the KLHDC subfamily; for example, it requires binding of GTP for activity [21].

**Table 1:** Sequence identity comparison of all KLHDC subfamilies. Sequences obtained and aligned in UniProt [14]. Where isoforms are known to exist, only the first isoform was used. Coloured based on % identity with high identity coloured green and low identity coloured red.

	Uniprot code		Q8N7A1	Q9Y2U9	Q9BQ90	Q8TBB5	Q5VTJ3	Q96G42	Q8IYD2	Q8IXV7	Q8NEP7	Q6PID8	Q7Z6M1	Q9UL63
		Protein name	KLHDC1	KLHDC2	KLHDC3	KLHDC4	KLHDC7A	KLHDC7B	KLHDC8A	KLHDC8B	KLHDC9	KLHDC10	RABEPK	MKLN1
% identity	Q8N7A1	KLHDC1	100											
	Q9Y2U9	KLHDC2	44.24	100										
	Q9BQ90	KLHDC3	25.29	25.08	100									
	Q8TBB5	KLHDC4	20.29	19.88	20.06	100								
	Q5VTJ3	KLHDC7A	16.67	15.07	16.85	15.43	100							
	Q96G42	KLHDC7B	19.17	16.25	21.32	17.42	38.27	100						
	Q8IYD2	KLHDC8A	16.67	14.69	18.64	13.73	24.91	21.16	100					
	Q8IXV7	KLHDC8B	17.27	14.29	15.6	17.07	20.77	20.95	46	100				
	Q8NEP7	KLHDC9	22.18	22.39	18.93	20.83	14.18	15.64	16.36	16.22	100			
	Q6PID8	KLHDC10	25.38	23.93	24.93	21.04	17.17	21.16	19.86	21.16	18.49	100		
	Q7Z6M1	RABEPK	23.12	25.3	20.66	18.32	12.94	18.62	18.45	16.54	18.55	23.95	100	
	Q9UL63	MKLN1	20.61	17.96	16.67	17.77	11.38	14.08	16.72	13.64	18.67	16.49	16.76	100

In comparison to the KLHL and KBTBD subfamilies, the KLHDC subfamily has a distinct lack of literature specifically pertaining to their physiological roles, structural domains and function. KLHDC2 is currently the only member of the KLHDC subfamily to have an X‐ray crystal structure, as well as extensive literature elucidating its function [22, 23, 24, 25, 26]. Despite the lack of data, what is known about KLHDC2, and other proteins containing related motifs, can be used to predict and understand the functions of other KLHDC members.

**Fig. 2:**
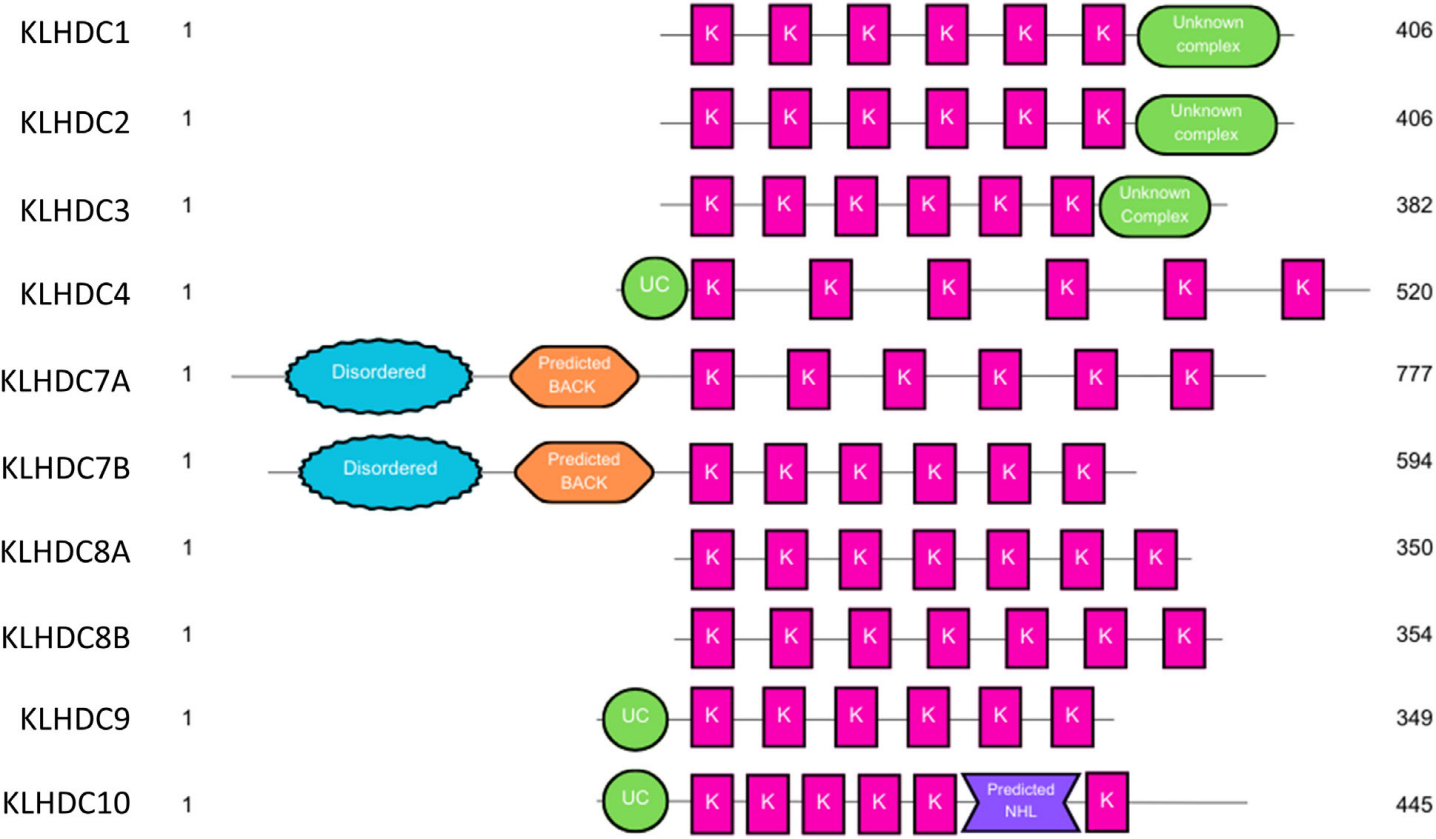
Schematic of structural differences between each KLHDC sub‐family member. In order top to bottom: KLHDC1, KLHDC2, KLHDC3, KLHDC4, KLHDC7A, KLHDC7B, KLHDC8A, KLHDC8B, KLHDC9, KLHDC10. Pink box: single Kelch repeat; green circle/oval: unknown complex; orange hexagon: predicted BACK complex; blue bubble: disordered region; purple inverted hexagon: predicted NHL repeat complex. Analysis was completed using alphafold structures; these structures, along with an X‐ray crystal structure of KLHDC2 (PDB: 8EBN, with other complexes removed), were used to analyse and compare the structural domains of the different family members.

As described above, KLHDC family proteins lack both the BTB and BACK domains found in the other Kelch subfamilies. However, multiple KLHDC members contain alternate domains, many with known function (Fig. 2). The variety of domains within the KLHDC subfamily is likely to contribute to functional differences [3] and suggests that there is extensive diversity in the biological functions of KLHDC members [4, 27].

**Fig. 3:**
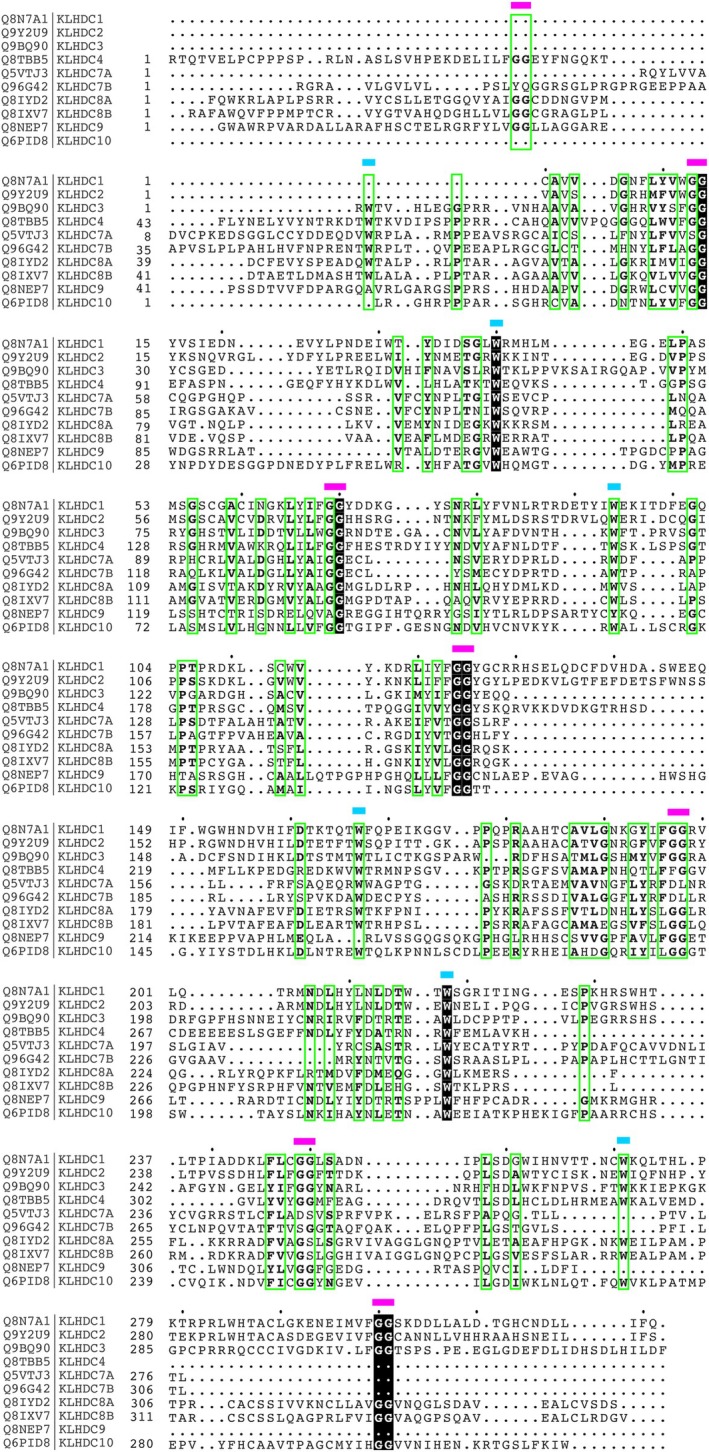
Alignment of Kelch β‐propeller of each of the KLHDC subfamily members. Protein sequences of human KLHDC subfamily members obtained from UniProt (codes within figure). Dots indicate every tenth amino acids of the protein sequence. Magenta bar: gly‐gly doublet; cyan bar: aromatic residue Tryptophan. White letters on black background: identical amino acids; bold letters in green box: highly similar amino acids according to physico‐chemical properties; standard letters: different amino acids. Alignment was performed using clustal omega [96].

Despite being of the same subfamily, there is low sequence identity (< 26%) among the KLHDC members (Table 1, Fig. 3), with some exceptions. For example, KLHDC1 and KLHDC2 share the highest sequence identity (44.24%), which aligns with the phylogenetic prediction that they are closely related. Similarly, KLHDC8A and KLHDC8B also share one of the highest levels of sequence identity (46%), alongside KLHDC7A and KLHDC7B, both of which are also phylogenetically predicted to be closely related. However, KLHDC7A, KLHDC7B, KLHDC8A and KLHDC8B also have the lowest sequence identity with the remaining members of the family (< 21%).

**Fig. 4:**
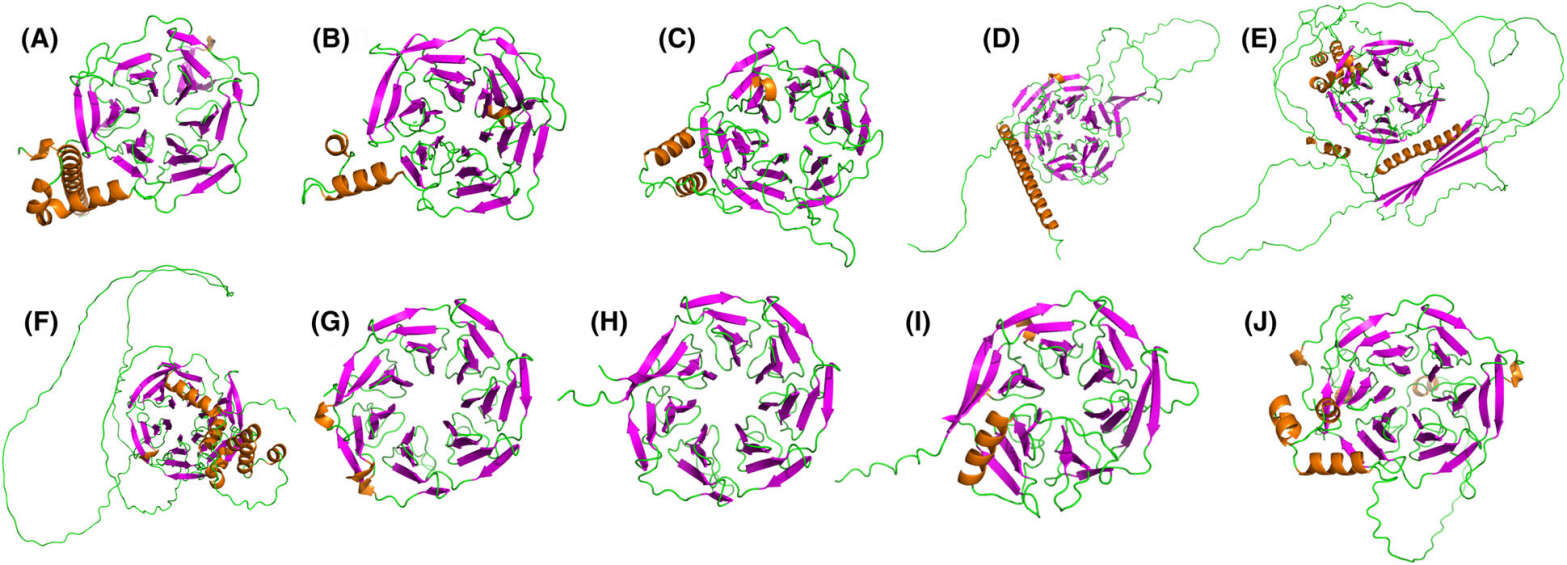
Predicted structures of KLHDC sub‐family. (A) Predicted crystal structure of KLHDC1 (AF_Q8N7A1‐F1), (B) experimental crystal structure of KLHDC2 from Scott *et al*. [22] (PDB: 8EBN), (C) predicted crystal structure of KLHDC3 (AF‐Q9BQ90‐F1), (D) predicted crystal structure of KLHDC4 (AF‐Q8TBB5‐F1), (E) predicted crystal structure of KLHDC7A (AF‐Q5VTJ3‐F1), (F) predicted crystal structure of KLHDC7B (AF_Q96G42‐F1), (G) predicted crystal structure of KLHDC8A (AF‐Q8IYD2‐F1), (H) predicted crystal structure of KLHDC8B (AF_Q8IXV7‐F1), (I) predicted crystal structure of KLHDC9 (AF‐Q8NEP7‐F1) and (J) predicted crystal structure of KLHDC10 (AF‐Q6PID8‐F1). All structures obtained from alphafold2 [13] and visualised in pymol (the pymol molecular graphics system, version 3.0 schrödinger, llc.).

Unsurprisingly, the region sharing the most identity between the subfamily members is within the Kelch β‐propeller domain. Bioinformatic analysis of all known Kelch‐repeat proteins [1] highlighted eight key conserved residues within each Kelch repeat, which allows for the formation of the β‐propeller [28] (Fig. 3). Specifically, these include a set of four hydrophobic residues, followed by a double glycine element separated from two aromatic residues, most often a tyrosine and tryptophan which was found to be conserved in 90% of the sequences [1, 28] (Fig. 3). For the KLHDC subfamily specifically, most members contain six Kelch repeats forming the β‐propeller Kelch domain, except for KLHDC8A and KLHDC8B which contain seven (Figs 2 and 4). Most of the members conserve the characteristic gly‐gly doublet within each Kelch repeat. However, there are varying degrees of conservation of the other conserved Kelch residues. Only one di‐glycine element is conserved across every member of the KLHDC subfamily, while six other conserved residues are observed at varying degrees across all members; except for members KLHDC4 and KLHDC7A which only have three and two di‐gly conserved elements, respectively, that align with the remaining family members (Fig. 3). It is also important to note that there is variable spacing between the glycine pair and conserved aromatic residues within each Kelch repeat, which does not always appear to form a consistent pattern [3].

## KLHDC structure

Only KLHDC2 has an experimentally determined structure (X‐ray crystal, Cryo‐EM, NMR) available [22, 23, 26]. There are currently 10 experimental structures of KLHDC2 in the protein data bank (Oct 2024) [29]. Eight of these are X‐ray crystal structures (PDB IDs: 8SGF, 8SGE, 8PIF, 8EBM, 8EBL, 6DO5, 6DO4, 6DO3), containing only the Kelch domain in complex with bound substrates. In 2023, an additional X‐ray crystal structure was determined, containing the full KLHDC2 structure in complex with bound adaptor proteins ELOB and ELOC (PDB: 8EBN). This structure revealed that the functional complex was likely to be a tetrameric structure, containing four KLHDC2 monomers with their adaptor proteins [22]. More recently, this tetrameric assembly was also supported by the determination of a Cryo‐EM structure (PDB: 8SH2) and adjacent biochemical studies which highlighted that this tetrameric structure can be modulated by substrate engagement [24]. This structure also depicts a flexible ‘lasso’ type region formed by amino acids 363–406, in which the end of this lasso (amino acids 400–406) can bind into the substrate binding pocket of another KLHDC2 within the same complex, and thus, the C‐terminal region is able to form its own substrate.

For the purposes of this review and to analyse the structural aspects of all other KLHDC members, alphafold2 [13] structures were obtained (Fig. 4). These structures, along with an X‐ray crystal structure of KLHDC2 (PDB: 8EBN [22], with other complexes removed), were used to analyse and compare the structural domains of the different family members. Additionally, interpro [17], a bioinformatics resource that provides a functional analysis of protein sequences by classifying them into families and predicting their domains and important sites, was also used to determine possible functions of the additional structural domains. These two sets of analyses are described below.

### Kelch domain

The intrablade loops which connect each β‐strand together are highly variable between each of the different KLHDC subfamily proteins. Additionally, the loops that connect each repeat to each other are also highly variable, with ranges in length predicted to be between 1 residue and ~ 35 residues. Since the conserved β‐propeller shape without these loops would be a flat, conserved surface, these differences in loop lengths lead to differences in the 3D shape of the proteins [1]. The centre of the Kelch domain is where the substrates are shown to bind [24, 30], and thus, these loops likely alter what other proteins each family member interacts with, leading to alternate functions [1, 26].

### Additional structural domains

Another feature of some KLHDC subfamily members is the presence of additional (non‐Kelch) structural domains, which may explain the wide variety of functions of KLHDC subfamily members.

Despite originally being defined as a KLHDC subfamily members, both KHLDC5 and KLHDC6 were predicted to contain BTB/POZ or BACK domains. Specifically, KLHDC5 was predicted to have a BTB/POZ domain between residues 5–72 and a BACK domain between residues 128–237, whereas KLHDC6 was predicted to contain a BTB/POZ domain at residues 28–128 and a BACK domain at residues 133–235 (Fig. 2). This prediction is supported by KLHDC5 also shown to interact with Cullin 3 (CUL3), which binds uniquely to BTB domains [12, 31]. KLHDC7A was also predicted to have a BACK domain between residues 430–505. It is also predicted to be extracellular, at residues 39–777, although there is no literature to support this (Fig. 2). KLHDC10 is predicted to have an NHL repeat at residues 281–382, a highly conserved six‐bladed β‐propeller, present in a wide variety of eukaryotic and prokaryotic proteins (Fig. 2). Like a Kelch repeat, an NHL repeat is involved in protein binding; however, it is also suggested to be important for RNA recognition [32].

The remaining subfamily members – KLHDC1, KLHDC2, KLHDC3, KLHDC4, KLHDC7B and KLHDC9 – had no predictions as to their additional structural motifs, despite having primary sequences which are predicted to fold outside of the Kelch domain (Figs 2 and 3). Meanwhile, KLHDC8A and KLHDC8B do not appear to have any distinct structural elements in addition to the Kelch β‐propeller domain (Figs 2 and 4). With so little known about their biology, it is unclear why these two members differ structurally and if they should be considered as a separate subgroup. Further research will have to elucidate this. While there are no predictions of the structural motifs of KLHDC2 or KLHDC3, research shows that the additional structural element – outside of the Kelch domain – is involved in interactions with the CRL2 complex, comprising of CUL2, RBX1, Elongin B (ELOB) and Elongin C (ELOC), in order to facilitate substrate binding and trigger protein degradation (Fig. 2) [22, 24, 30, 33, 34].

## Physiological roles of KLHDC family members

### Localisation and expression

The KLHDC subfamily is distributed throughout the cell with some members shown to localise to the nucleoplasm (KLHDC2, KLHDC3, KLHDC9, KLHDC10) [35, 36], nuclear speckles (KLHDC7A) [35], cytoplasm (KLHDC3) [7, 33, 37] and nucleoli (KLHDC4) [35]. This is suggestive of a role related to DNA and RNA biological processes. Some members are shown to reside in the cytosol such as KLHDC1 and KLHDC8B [35]. KLHDC1 was shown to be cytoplasmic, despite being fused to strong nuclear localisation signals, leading to the suggestion that it must be sequestered to the cytosol *via* unknown partner protein(s) [38]. KLHDC7B is located within the plasma membrane [35], which supports the prediction of this protein having an extracellular domain. The specific cell localisation of KLHDC8A remains unknown. Finally, in HeLa cells, KLHDC8B was shown to only be expressed in mitotic cells, concentrating in the region where the cells separate, suggesting a role in cytokinesis [39].

Where the tissue localisation and expression are known, most KLHDC subtypes are widely expressed [35] (Table 2). However, some members have been shown to be expressed at high levels in tissues involved in reproduction, specifically the testis (KLHDC1, KLHDC3, KLHDC8B and KLHDC10) [40], epididymis (KLHDC8B) [40], seminal vesicle (KLHDC8B) [40] and endometrium and breast (KLHDC4) [40], highlighting their potential role in cell division, specifically meiotic recombination [41]. Additionally, KLHDC subfamily members may also play a potential role in regenerative cells since there is also a high expression of KLHDC1, KLHDC2 and KLHDC3 in skeletal muscle [7, 38] and liver cells [40] (Table 2).

**Table 2:** The localisation, expression and physiological roles of KLHDCs. Individual references are within the table.

Gene name	Localisation	Expression	Function
KLHDC1	Intracellular – cytosol [36, 38]	Widely expressed – high levels in skeletal muscle, pancreas and liver [38]	Enables ubiquitin ligase‐substrate adaptor activity [60] Destabilises truncated selenoproteins [60] Involved in ubiquitin‐dependent protein catabolic process *via* the C‐end degron rule pathway [60]
KLHDC2	Intracellular – nucleoplasm, nuclear membrane, nuclear bodies [36, 38]	Widely expressed – high levels in liver, testis [36], skeletal muscle, heart [38]	Substrate‐recognition component of a Cul2‐RING (CRL2) E3 ubiquitin‐protein ligase complex of the DesCEND (destruction *via* C‐end degrons) pathway [26, 43, 44] It may also act as an indirect repressor of CREB3‐mediated transcription by interfering with CREB3‐DNA‐binding [76] Can engage the substrate binding domains of another protomer using its C‐terminal Gly‐Ser motif to mimic a degron [22]
KLHDC3	Intracellular – nucleoplasm [36]	Widely expressed – high levels in testis and skeletal muscle [7, 36]	Involved in the activation of the V(D)J recombination [7] In mice, this gene is found to be expressed specifically in testis [7] CRL2 adaptor part of the ubiquitin‐proteasome system and is known as a Cullin2‐RING E3 ubiquitin ligase (CRL2) complex adaptor that recognises specific protein substrates and targets these for degradation by the CRL2 ubiquitin proteasome system [44, 53] Targets proteins containing glycine at their C‐terminal residues [26, 44] Mediates ubiquitination and degradation of truncated SELENOV and SEPHS2 selenoproteins produced by failed UGA/Sec decoding, which end with a glycine [26, 43]
KLHDC4	Intracellular – nucleoli and kinetochore [36]	Unknown	Unknown
KLHDC7	Intracellular – specific location unknown [36]	Unknown – however KLHDC7A is thought to express mainly in the kidney [36]	Believed to be responsible for protein coding [77]
KLHDC7A	Intracellular – nuclear speckles [36]		
KLHDC7B	Intracellular – plasma membrane [36]		
KLHDC8	Intracellular – specifics unknown [36]	Unknown – however KLHDC8A is thought to express in the ovary [36]	KLHDC8B is understood to be required for mitotic integrity and maintenance of chromosomal stability. Additionally, it is involved in pinching off the separated nuclei at the cleavage furrow and in cytokinesis I [41] and is thought to participate in cytokinesis, where reduced expression leads to formation of binucleated cells [39]
KLHDC8A	Intracellular – specifics unknown [36]		
KLHDC8B	Intracellular – cytosol [36]		
KLHDC9 (previously known as KARCA1)	Intracellular – nucleoplasm, nucleoli, nucleoli rim, mitotic chromosome [36]	Widely expressed – mainly within testis [36, 78]	Believed to enable cyclin binding [78]
KLHDC10	Intracellular – nucleoplasm [36]	Widely expressed – high levels in testis and brain	Participates in oxidative stress‐induced cell death through MAP3K5 activation [71] Inhibits PPP5C phosphatase activity on MAP3K5 [71] Specifically recognise proteins with a proline‐glycine at the C terminus just as KLHDC3 [44]

### The KLHDC subfamily role in the ubiquitination pathway

**Fig. 5:**
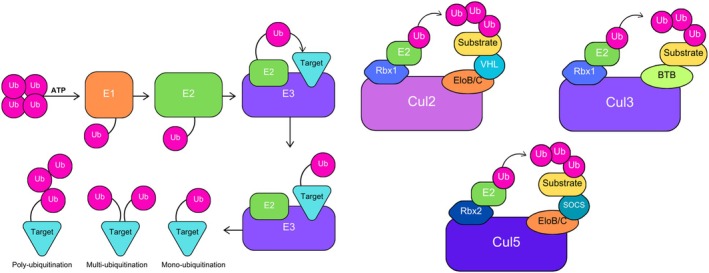
Schematic of KLHDC role in ubiquitination. Left panel: Simplified schematic of the cascade process for ubiquitination to occur. E1 activates Ub through a thioester bond in an ATP‐dependent process. Then, the activated Ub is ‘transferred’ to an E2 enzyme's active‐site cysteine. Finally, an E3 ligase recognises the E2 complex and transfers Ub from E2 to the target substrate. Based on how Ub binds to the substrate, ubiquitination can be mono‐, multi‐, or poly‐ubiquitination. Right panel: Schematic of three E3 CRL complexes specific to this review; Cul2, Cul3 and Cul5 recruit specific adaptor proteins (Elongin B/C for Cul2 and Cul5, BTB protein for Cul3) and receptor proteins (VHL‐box for Cul2, SOCS for Cul5) to form CRL E3 ubiquitin ligases with the RING protein (Rbx1/2). These ligases transfer ubiquitin from Rbx1/2‐bound E2 to substrate proteins. Figures adapted from [45].

All members of the KLHDC family are thought to be substrate receptors of E3 ligase complexes [3] responsible for targeting and tagging misfolded or otherwise damaged proteins for degradation [24]. The degradation of these proteins is generally happening through two methods, the ubiquitin proteasomal system and/or autophagy [24, 42], which are both essential for the maintenance of homeostasis. As the name suggests, the ubiquitin proteasomal system (UPS) is the term for the cascade of events leading to the ubiquitination of specific proteins, that is, where ubiquitin is ‘transferred’ onto proteins by covalent conjugation. This process requires a set of three enzymes: E1s (ubiquitin activating enzymes), E2s (ubiquitin conjugating enzymes) and E3 ligases which selectively attach ubiquitin to lysine, serine, threonine or cysteine residues to the specific substrates in need of degradation [42] (Fig. 5). Specifically, KLHDC proteins are specifically essential for the destruction *via* C‐end degrons (DesCEND) mechanism of ubiquitination. This mechanism specifically targets substrates with specific C‐terminal motifs for degradation [26, 43, 44].

E3 ligases are generally categorised according to their structure and function. These form complexes consisting of a scaffold subunit, a catalytic subunit and an adaptor protein. The complex then binds to a group of proteins that are responsible for substrate recognition or the substrate recognition receptor (SRR) [42, 45]. It is thought that Kelch proteins act as SRRs in the cullin‐RING (Really Interesting New Gene) ubiquitin ligase (CRL) network [46, 47]. CRLs comprised four major components: Cullin proteins (CUL1, CUL2, CUL3, CUL4A/4B, CUL5 and CUL7) that act as the central scaffold which binds to a RING‐box protein (in most cases RBX1 or RBX2) which then binds to a E2 ubiquitin conjugating enzyme at the N‐terminal and an adaptor protein‐substrate receptor complex which recognises the target protein and connects the SRRs back to the Cullin at the C‐terminal [42, 45]. Each CRL subfamily has a distinct set of adaptors or substrate recruiters. Of specific interest to this review are CUL2 and CUL5, which both interact with Elongin B and Elongin C (ELOB/C), and CUL3, which uses BTB/BACK/POZ (broad complex, tramtrack and bric‐à‐brac/poxvirus and zinc finger)‐domain proteins as both adaptor and substrate receptor [45] (Fig. 5). It is these ELOB/C and BTB/BACK/POZ complexes which recruit BC‐box proteins to act as substrate receptors [34, 48].

Since the KLHDC subfamily members lack BTB‐BACK domains, they are required to recruit BC‐Box proteins to enable efficient ubiquitination. The BC‐box has a consensus sequence of (S,T,P)LXXX(C,S,A)XXXΦ, where X represents any amino acid, and Φ represents the hydrophobic amino acids [49]. It is then through these BC‐box proteins that the selectivity of substrates occurs through the recognition of specific short peptide motifs termed degrons [50, 51]. Emerging research has indicated that some KLHDC subfamily members such as KLHDC2 and KLHDC3 utilise the CRL2 complex to facilitate these previously described steps of ubiquitination [26, 30, 43, 44, 52, 53, 54].

### KLHDC2

The most well‐studied member is KLHDC2, which has been shown to be a substrate‐recognition component of the Cul2‐RING E3 ubiquitin‐protein ligase complex which functions in the DesCEND pathway. Thus, the C‐terminal region of CUL2 binds to RING‐box protein 1 (RBX1) and the N‐terminal region binds to Elongin B/C, followed by the BC‐box proteins – VHL (Von Hippel–Lindau) being the prototype – to form this complex [43, 48]. KLHDC2 recognises specific sequences located near the C‐terminal regions of target proteins, known as C‐degrons; the recognition of these C‐degrons then leads to their ubiquitination and degradation [26, 43, 44]. These C‐degrons bind within the substrate‐binding pockets, located at the top of the β‐propeller of the KLHDC proteins. Specifically of note is the di‐glycine motif at the C‐terminal [22, 26], referred to as a di‐Gly degron, which is present in most substrates. This has been highlighted by crystal structures of KLHDC2 in complex with an early‐terminated selenoprotein – SelK and SelKS – C‐end degron peptide (PDB code: 6DO3 [26]). This selenoprotein SelK incorporates a selenocysteine (Sec), which is encoded by the stop codon UGA [55]. This codon is interpreted as Sec due to a Sec insertion sequence element in the 3′ untranslated region (UTR) of SelK mRNA, facilitating the synthesis of the complete protein. When selenium is deficient, the absence of Sec‐transfer RNA leads to premature translation termination [56]. Recent research indicates that the truncated SelK protein produced under these conditions possesses a C‐terminal di‐Gly degron, marking it for CRL2‐KLHDC2‐mediated proteasomal degradation [26]. Several additional X‐ray structures such as a peptide derived from EPHB2 (PDB code: 8EBL [22]) have revealed that KLHDC2 is also able to bind with other C‐degron containing proteins.

The 3D structures, along with extensive biochemical studies, have shown that a specific Gly‐Gly motif is essential at the C‐terminal. However, in attempting to resolve how the KLHDC2‐ELOB/C substrate receptor is regulated [22], these studies were also able to reveal that KLHDC2 (which terminates at the C‐terminal end with a Gly‐Ser [24]) can mimic a di‐Gly degron with its C‐terminal 401‐NNTSGS‐406 C‐degron mimic. This degron mimic has been shown to bind at lower affinity than the substrates, potentially due to the additional atoms of Ser406, causing Trp270 into a slightly less energetically favourable orientation [24]. Interestingly, structural data suggested that KLHDC2's activity could be self‐regulated [22]. Using this degron mimic, KLHDC2 forms a complex where one KLHDC2 C terminus binds to another KLHDC2 protein, thus self‐regulating its activity [22, 24].

KLHDC2 has recently had ligands developed against it which provided pharmacological evidence that KLHDC2 could be a good PROteolysis TArgeting Chimeras (PROTAC) target for specific protein degradation [23, 24, 25]. PROTACs are designed as bifunctional molecules that consist of two main components. One part of the molecule binds to the target protein you want to degrade, and the other part recruits an E3 ubiquitin ligase, in this case KLHDC2, which tags the target protein for destruction. This approach allows for targeted degradation of proteins that are often difficult to inhibit with traditional small molecules, offering a new strategy for drug development, especially in cancer and other diseases.

### KLHDC3 and KLHDC10 are also classified as CUL2‐RING E3 ligases

KLHDC3 has been often studied concurrently with KLHDC2 [22]. Like KLHDC2, it is a substrate‐recognition component of a CRL2 E3 ubiquitin‐protein ligase [44, 53]; however unlike KLHDC2 substrates which contain the diGly degron as discussed above, a study by Koren *et al*. [44] discovered that only the C‐terminal glycine residue is absolutely critical for degradation; KLHDC3 recognises substrates through interaction with other exposed C‐terminal degrons such as ‐RG, ‐KG or ‐QG motif. KLHDC3 was originally identified by its ability to distinguish defective selenoproteins by their C‐terminal residues. Thus, it can mediate ubiquitination and degradation of truncated SelS and SEPHS2 selenoproteins produced by failed UGA/Sec decoding, which also end with a glycine [26, 43]. Moreover, like KLHDC2, KLHDC3 is able to form complexes with both ELOC and ELOB [22]. Notably, like KLHDC2's GlySer autoinhibitory motif, KLHDC3 contains a C‐terminal HisGly motif and can form a similar tetrameric structure as KLHDC2 [22] although it is unclear whether it has the same autoinhibitory function. On the other hand, while KLHDC10 can form a complex with ELOB/C akin to its other subfamily members, KLHDC2 and KLHDC3, it was not able to form a tetrameric structure, instead remaining monomeric [22]. Since KLHDC10 has a C‐terminal ending in Leu‐Lys, this finding aligns with the fact that the C‐terminal sequence has diverged from the cognate degron [22], potentially preventing the engagement of its substrate‐binding pocket [24]. However, alphafold2 [13] models suggest the N terminus of KLHDC10 may fold back on itself and bind to the central substrate pocket of KLHDC10. The N terminus has Alanine in positions 3 and 4, and a modelled Ala‐tail of at least 5 consecutive Alanine's with a short GFP‐derived sequence was found to consistently bind to KLHDC10 [57]. Although these interactions are yet to be further validated and understood, they could suggest a similarity with KLHDC2's autoinhibition and a similar mechanism for this protein to regulate its activity.

### KLHDC1 binds Cullin‐5 for ubiquitin activity

Like CUL2, CUL5 also interacts with ELOB/C. However, unlike CUL2 which tends to form a complex with VHL box (BC box and CUL2 box)‐containing proteins (named after VHL the prototype substrate receptor of CUL2), CUL5 usually forms a complex with SOCS box (BC box and CUL5 box) containing proteins of which SOCS2 is the prototype [45]. The interaction of CUL2 vs. CUL5 with ELOB/C is subtle and purely due to the difference in the CUL2 or CUL5 boxes of the proteins they each recruit [45, 58, 59]. KLHDC1 interacts with CUL5, forming a CUL5‐RING ubiquitin ligase complex, which is thought to target proteins for ubiquitination and degradation in a manner similar to CUL2‐KLHDC2 [48]. Additionally, KLHDC1 has recently been shown to target the same di‐Gly degron and destabilise truncated selenoproteins [60].

### KLHDC4, KLHDC7A, KLHDC7B, KLHDC8A, KLHDC8B, KLHDC9

Limited research exists for KLHDC4, which suggests that it may not be able to interact with CUL2 or CUL3 [61]. Structural analysis performed by Lian *et al*. [61] suggested that KLHDC4 lacks the consensus modules required to interact with Cullin proteins, which supports the concept that KLHDC4 may exert its function through other means. No current information regarding their function within the ubiquitin system exists for KLHDC7A, KLHDC7B, KLHDC8A, KLHDC8B and KLHDC9.

### Non‐ubiquitin‐related KLHDC functions are mostly related to protein coding and cell cycle regulation

Despite their large role in the ubiquitin pathway, there are some literature studies which support alternate roles for KLHDC family members. There is some evidence that KLHDC3 and KLHDC8B may be involved in cell cycle regulation [7, 41]. KLHDC3 is structurally akin to recombination activating gene 2, a protein involved in the activation of the V(D)J recombination, thus has a proposed role in meiotic division [7], though this was based solely on the β‐propeller of RAG2 which at the time of this publication was the most closely related structure. However, since KLHDC3 primarily localises to pachytene spermatocyte cytoplasm and meiotic chromatin [7], this could suggest that KLHDC3 plays a role in meiotic division. KLHDC8B is involved in pinching off the separated nuclei at the cleavage furrow and in cytokinesis 1, ultimately thought to protect cells against mitotic errors, centrosomal amplifications, micronucleus formation and aneuploidy [41].

## Known protein–protein interactions (PPI) and substrates of KLHDCs

Although there is limited literature on the KLHDC subfamily and their physiological roles, as well as a lack of experimentally determined crystal structures, predicted PPIs and substrate binding can provide insights into the potential physiological functions of different KLHDC proteins. The functions of most KLHDC subfamily members have been derived from research not specific to KLHDC subfamily, and therefore, we have collated these data (Table 3) along with known/predicted protein interactions to better understand the roles of the subfamily.

**Table 3:** Known Protein–protein interactions of KLHDC family members. Protein–protein interaction (PPI) data collated from various databases (BioGrid [79], IntAct [80] and STRING [81]) using default parameters. Each data set was analysed, and the top five known PPIs based on their highest amount of evidence surrounding their interactions listed. Shown are the eight C‐terminal amino acids of each protein, anticipated to be the C‐degron each KLHDC protein member interacts with.

Gene name	Number of protein–protein interactions per database			Top 5 known PP interactions (in no order)	C‐terminal sequence
	BioGrid	IntAct	STRING		
KLHDC1	3	2	33	CUL5 [82]	INTFIYMA
				TCEB2 [82]	SANEQAVQ
				SPPL3 [83]	SSSRFLEV
				ELOB [84]	SANEQAVQ
KLHDC2	133	64	61	CUL2 [54, 85]	ADEYSYVA
				CUL5 [86]	INTFIYMA
				APPBP2 [54]	QNVEGPSC
				RBX1 [84]	WEFQKYGH
				KLHDC3 [54, 60]	RPIVSSHG
KLHDC3	172	56	38	CUL2 [60]	ADEYSYVA
				ELOB [54]	SANEQAVQ
				RBX [60]	WEFQKYGH
				APPBP2 [43]	QNVEGPSC
				KLHDC2 [60]	GSNNTSGS
KLHDC4	41	20	26	ARMC6 [87]	GQRGNLAP
				CROT [85, 86]	QLMNSTHL
				AHR [85]	DLTSSGFL
				CES2 [85]	PEERHTEL
				ZCCHC14 [88]	AESLDSTD
KLHDC7A	2	–	5	KLHDC10 [3]	QGLIERLK
				ORF9B	EFVVVTVK
				IGSF21 [89]	LTVILELT
				SLC25A45 [90]	EYLLRWWG
				PPM1J [90]	LGGPGSYS
KLHDC7B	27	72	3	CLM2 [91]	PQWAPPGR
				OR4F21 [92]	LVIYKKIS
				ZNF487 [92]	VDFTQEEW
				EPHB6 [93]	RQQGSVEV
				A1CF [94]	RGDGYGTF
KLHDC8A	20	18	10	PEX7 [85]	PACLTIPA
				TXNDC9 [86]	DSDSDDD
				CCT2 [86]	RVPDHHPC
				CCT3 [86]	GAPDAGQE
				CCT4 [86]	IDDVVNTR
KLHDC8B	12	4	10	CCT2 [85]	RVPDHHPC
				CCT3 [85]	GAPDAGQE
				CCT5 [85]	RKPGESEE
				CCT7 [85, 86]	RGRGRPH
				IMPDH1 [85, 86]	HSYEKRLY
KLHDC9	37	5	10	CA3 [85]	RVVRASFK
				CAV3 [85]	KVVLRKEV
				CCDC134 [85]	ISRSQSEL
				CEACAM8 [85]	VLARVALI
				CKB [85]	DDLMPAQK
KLHDC10	74	20	10	CUL2 [44]	ADEYSYVA
				ELOB [44]	SANEQAVQ
				APPBP2 [44]	QNVEGPSC
				KLHDC3 [44]	RPIVSSHG
				KLHDC2 [44]	GSNNTSGS

### Protein–protein interactions between KLHDC2, KLHDC3 and KLHDC10 and CUL2/ELOC/ELOB are supported by experimental research

PPIs of KLHDC2, KLHDC3 and KLHDC10 are among the highest predictions of the family. Discussed previously, KLHDC2, KLHDC3 and KLHDC10 have shown to be SSRs of CUL2, as well as ELOB and ELOC, and thus, the prediction of interactions between these KLHDC subfamily members and CUL2 is unsurprising. However, there is also a predicted interaction between CUL5 and KLHDC2 (Table 3). While there is currently a lack of experimental data to support this interaction [34], we know that KLHDC1 and KLHDC2 share some sequence identity (17.8%), and like KLHDC1, KLHDC2 is able to recognise the diGly degron at the C terminus of truncated SELENOS, as previously described [60]. Thus, although it is possible that KLHDC2 interacts with CUL5, experimental validation would be required to confirm this prediction.

### KLHDC‐substrate relationships

The information gleaned from KLHDC2 can be used to understand the role of other KLHDC subfamily members. As previously discussed, KLHDC1, KLHDC2 and KLHDC3 have been shown to recognise specific degrons (diGly and single Gly at the C terminus, respectively), while KLHDC3 recognises substrates through interaction with other exposed C‐terminal degrons, such as ‐RG, ‐KG or ‐QG motif [44].

As aforementioned, the early termination of both SelK and SelS proteins rendered their C‐end degron to be ‐Gly‐Gly, allowing KLHDC2 to target these proteins. The C‐terminal peptide of these truncated selenoproteins binds to the centre of KLHDC2 between its propeller blades which form a binding pocket [26]. KLHDC1 has also been determined to bind to truncated SelS [60]. In addition, from a global protein stability assay, KLHDC3 was found to target both UGA‐terminated selenoproteins SelS and selenophosphate synthetase 2 (SEPHS2) [62].

Only a handful of full‐length proteins have been identified and validated as targets of KLHDCs. These include PTOV1, PDP2 and MIC19 for KLHDC2 and PPP1R15A, USP49, TCAP, p14ARF and TSPYL1 for KLHDC3 [43, 44, 54]. A study investigating the C‐degron pathway provides a comprehensive list with 20 KLHDC2‐binding full‐length proteins identified through GST pull‐down and mass spectrometry, most of which are diGly‐ending, although not all have been verified [54].

In human cells, KLHDC3 binds p14ARF and targets it for degradation in a CRL2 complex‐dependent manner through the recognition of p14ARF's C‐terminal degron [33, 63]. This degron is, however, not conserved in mice. It has also been shown that KLHDC3 interacts with and contributes to the degradation of c‐Myc in tandem with Fbxw7, functioning in the regulation of cell growth and tumour formation [37].

Recent studies on KLHDCs have emphasised that the conformation of the substrate is the determining factor when it comes to their binding tendencies. KLHDC3 recognises the C‐degron sequence (−RGRG) of the herpesvirus UL49.5 protein which acts in ER‐associated degradation pathways. A mutation leading to the protein having a similar conformation preserves crucial interactions, whereas a mutation that can significantly alter the C‐end's conformation results in the loss of the substrate's ability to adopt in the KLHDC3 binding cleft [64]. A related study suggested the importance of an arginine residue being the fourth amino acid from the C terminus and a positively charged residue located directly upstream of the C‐terminal Gly (at the penultimate position); however, more evidence will be necessary to support this [65]. This is also supported by others who established the crucial role of either a charged or polar residue, of which positively charged residues fit, as the penultimate amino acid for a degron to be recognised [54].

There is limited information on the rest of the KLHDC family's interacting substrates. KLHDC8A binds Chaperonin‐Containing TCP1 (CCT) and is speculated to act as an adapter that facilitates CCT‐α‐tubulin interaction which ultimately contributes to the regulation of tubulin biogenesis [66]. KLHDC9, also known as KARCA1, interacts with the cyclin A1‐CDK2 complex [67]. Finally, aside from the previously discussed potential of KLHDC10 to have interactions that are reminiscent of KLHDC2's C‐degron mimicry, this member of the KLHDC family generally recognises substrates enriched with ‐WG, ‐PG and ‐AG endings [44]. KLHDC10 binds to the C‐terminal phosphatase domain of protein phosphatase 5 (PP5). This in turn suppresses its phosphatase activity, although this function is not dependent on its role as a substrate receptor of the CRL2 complex [68]. KLHDC10 also binds to homopolymeric Alanine tails (with at least four Ala residues) and Alanine‐rich C‐degrons, mediating their degradation in the ribosome‐associated quality control pathway [57, 69].

## Diseases related to the KLHDC family

### Cancer

Unsurprising based upon their role in the ubiquitination pathway, some KLHDC subfamily members have been reported to play a role in the development of several tumours (Table 2). However, their precise relationship remains largely unknown. In non–small‐cell lung cancer (NSCLC), high KLHDC3 expression is associated with poorer survival rates [53]. A CRISPR/Cas9 knockout of KLHDC3 significantly reduced lung cancer cell growth and suppressed tumour growth *in‐vivo* [53]. The study also found that KLHDC3 interacts with p14ARF through its β‐propellor, recruiting p14ARF to the CRL2 complex and promoting its N‐terminal ubiquitylation [33]. This process leads to p14ARF degradation *via* the UPS, thereby promoting NSCLC proliferation [33, 53]. KLHDC3 may also play roles in other types of tumours such as testicular and ovarian tumours [33, 53]. Inhibiting KLHDC3 with peptides or small molecule inhibitors could offer a promising therapeutic strategy for cancers overexpressing KLHDC3, although no inhibitors currently exist. Similarly, KLHDC4 has been implicated in nasopharyngeal cancer by suppressing apoptosis, and CRISPR/Cas9 knockout of KLHDC4 has shown reduced tumour growth and migration [61], making it another potential therapeutic target, despite the lack of available inhibitors. KLHDC7A, KLHDC7B and KLHDC9 have been associated with breast and ovarian cancer, where their expression correlates with cancer cell proliferation, but these findings are based on transcriptome‐wide studies and require further experimental validation.

Both KLHDC8A and KLHDC8B have been implicated in glioma cell apoptosis and classical Hodgkin lymphoma, respectively. KLHDC8A is significantly overexpressed in high‐grade gliomas compared to low‐grade and normal brain tissues. This expression is correlated with poor prognosis in patients [70]. The study showed that upregulation of KLHDC8A promotes glioma cell proliferation, migration and invasion while inhibiting apoptosis [70]. Additionally, the introduction of lactate induces KLHDC8A, linking metabolic changes to tumour progression [70]. Mutations of KLHDC8B were shown to be associated with an increased risk of developing classical Hodgkin lymphoma [70]. The study specifically highlights that these mutations could disrupt normal cell division processes, which lead to chromosomal instability. In addition to this finding, the study also found that KLHDC8B regulates proteins including Bcl2/BAX and p21/CDK2, all thought to indirectly influence the mitotic checkpoint, further highlighting the importance of maintaining genomic stability [70]. Finally, KLHDC10 has been implicated in the regulation of oxidative stress‐induced ASK1 activation by suppressing PP5 [71]. It is thought that KLHDC10 possesses two functions, one as a SRR as described above and another as a signal regulator through its interaction with PP5 [71]; however, associations with cancer are not well documented.

Like KLHDC3 and KLHDC4, the research conducted on the other family members indicates that targeting this subfamily could be a potential therapeutic strategy for various KLHDC‐overexpressing cancer types; however, potential inhibitors are not well documented. Conversely, targeted protein degradation using proteolysis targeting chimeras (PROTACs) is currently being explored as a therapeutic strategy for targeting cancer‐related oncoproteins that cannot be treated with classical small molecule inhibitors. As such, KLHDC2 is emerging as a novel powerful degrader, and the hope is that through the development of potent small molecule ligands that recruit KLHDC2 to the target protein, it will be able to specifically degrade proteins involved in cancer [72].

### Neurological diseases

The KLHDC subfamily has also been implicated in neurological functions and disorders [46, 73, 74, 75] (Table 2). KLHDC1 and KLHDC3 may be involved in neural differentiation and have been associated with psychiatric disorders, particularly schizophrenia [46, 74], suggesting a role in the development and function of the nervous system; however, this is not well defined. As discussed, KLHDC8A is linked to glioma [70], indicating a possible role in neurological malignancies. KLHDC10, known for its role in oxidative stress response [71], may be relevant to neurodegenerative diseases, but further research is required to establish its direct implications. However, for the remainder of the KLHDC subfamily – KLHDC1, KLHDC3, KLHDC4, KLHDC7A and 7B, KLHDC8B and KLHDC9 – direct associations with neurological disorders are not well documented, and comprehensive research is still required to fully understand their roles, if any.

## Conclusion

The KLHDC subfamily has gained significant interest in recent years, especially KLHDC2 which has been highlighted as an excellent E3 ligase substrate adaptor suitable for use as a PROTAC drug. The new knowledge gathered from these recent structural and biochemical advances for KLHDC2, along with predictive software such as alphafold and interpro, has allowed for a greater understanding of the other KLHDC family members. This review consolidates all the known structure and functional roles of these family members. There has been some debate in the field over which proteins should be included in the KLHDC family. The structural analysis conducted in this review in combination with the supporting literature suggested that there should only be 10 family members: KLHDC1, KLHDC2, KLHDC3, KLHDC4, KLHDC7A and 7B, KLHDC8A and 8B, KLHDC9 and KLHDC10. With the emergence of PROTACs in the drug discovery field, a greater understanding of the KLHDC family members may lead to the design of novel potent PROTAC reagents and allow diverse targeting of various pathology‐related proteins.

## Author contributions

CP created the models and the figures and wrote the first draft. PAVB wrote the section on substrates. JQT conducted the protein domain analysis. JKH conceptualised this manuscript and conducted extensive editing. In conjunction with all authors, PAR, MFS and CRW edited and reviewed the manuscript.
